# Attitudes and Willingness of Cardiothoracic Group Physicians in the Cardiovascular and Radiology Departments toward the Adjuvant Use of CT-Derived Fractional Flow Reserve in the Diagnosis of Coronary Artery Disease

**DOI:** 10.5334/gh.1477

**Published:** 2025-10-06

**Authors:** Xi Tian, Bingzhen Jia, Xusheng Lou, Dong Li, Zhang Zhang

**Affiliations:** 1 Department of Radiology, Tianjin Medical University General Hospital, Tianjin, 300052, China; 2 Peking University Sixth Hospital, Peking University Institute of Mental Health, NHC Key Laboratory of Mental Health (Peking University), and National Clinical Research Center for Mental Disorders (Peking University Sixth Hospital), Peking University, Beijing, 100191, China; 3 Department of Cardiovascular Medicine, Beijing University of Chinese Medicine Third Affiliated Hospital, Beijing, 100029, China

**Keywords:** Attitude, physicians, Computed Tomography, Fractional Flow Reserve, Coronary Artery Disease

## Abstract

**Background:**

The diagnosis of coronary artery disease (CAD) has traditionally relied on invasive coronary angiography (ICA), a method with inherent risks. As a noninvasive technique, computed tomography-derived fractional flow reserve (CT-FFR) can integrate both anatomical and functional assessments of the coronary arteries, identifying hemodynamically significant stenosis and thereby reducing unnecessary invasive procedures. Although its clinical value has been demonstrated, its widespread clinical adoption is constrained by physician perception.

**Objective:**

To quantify the professional attitudes and willingness to adopt CT-FFR for clinical application among cardiologists and radiologists, and to identify the key determinants influencing their positivity.

**Methods:**

A cross-sectional survey was conducted from May to June 2023 across five provinces and cities in China. Data were collected from 265 cardiothoracic physicians using a validated, structured questionnaire (Cronbach’s α = 0.884). The questionnaire assessed two core dimensions using a five-point Likert scale: “Attitude” (15 questions) and “Willingness” (eight questions). Higher scores indicated more positive attitudes or willingness.

**Results:**

The survey was completed by 265 physicians, with overall attitudes being positive. The median scores for the attitude and willingness dimensions were 51 (interquartile range: 48, 55) and 31 (interquartile range: 29, 32), respectively, with a significant positive correlation between them (r = 0.571, p < 0.001). While over 60% of physicians acknowledged that CT-FFR could prevent unnecessary invasive procedures, 38.1% still expressed concerns about its diagnostic accuracy. Logistic regression analysis showed that physicians working in specialized cardiovascular hospitals held more positive attitudes (OR = 3.085, p = 0.017). Multivariable analysis further confirmed that a positive attitude was the strongest independent predictor driving willingness to adopt (OR = 6.280, p < 0.001).

**Conclusion:**

Participants’ belief in the development potential of CT-FFR was positively associated with their willingness to learn, receive training, consider improvements, and participate in clinical research involving CT-FFR.

## Introduction

Coronary artery disease (CAD) is a widespread cardiovascular condition primarily attributed to the development of lesions in the coronary arteries (1,2). In recent years, the prevalence and mortality rates of CAD have shown an upward trend (3,4) coinciding with shifts in the social environment and lifestyle habits. This disease is characterized by the narrowing or obstruction of coronary arteries, leading to myocardial ischemia and angina (5). Consequently, early diagnosis and appropriate treatment are of utmost importance in preventing severe complications, including myocardial infarction. Traditionally, coronary angiography has been the invasive method of choice to assess coronary artery stenosis (6). However, this procedure carries inherent risks and inconveniences.

In recent years, noninvasive alternatives like coronary computed tomography angiography (CCTA) and computed tomography-derived fractional flow reserve (CT-FFR) have gained significant attention as diagnostic tools for CAD (7,8). CT-FFR, a noninvasive technique utilizing CT and computational fluid dynamics simulations, allows for the evaluation of coronary artery stenosis parameters and assessment of hemodynamically significant stenoses without the necessity of additional testing and radiation exposure, aiding in the determination of stenosis severity and its effect on myocardial blood supply (9). Despite the potential clinical benefits of CT-FFR in coronary artery disease diagnosis (7,8), its widespread adoption and application in clinical practice face challenges. Although CT-FFR is not currently included in practice guidelines in China, the PLATFORM study showed that CT-FFR could improve decision-making, patient management, and resource utilization (10). The FORECAST trial showed that CCTA with off-site CT-FFR analysis reduced the need for invasive coronary angiography (11). CT-FFR can now be included on-site to improve risk prediction (12,13). The ADVANCE study showed that CCTA without CT-FFR resulted in >50% of the patients needing retesting (14). The CT-FFR CHINA study supported CT-FFR as a valuable tool for the assessment of coronary calcification (15). On the other hand, although CT-FFR can be performed using modern CT scanners equipped with the appropriate software, there may be some concerns regarding the direct (image interpretation and diagnosis) and indirect (related to ensuing decisions about patient management) costs of the examination in relation to the benefits, since health economics studies are lacking. The TARGET trial showed that on-site CT-FFR reduced the need for invasive angiography but increased revascularization without improvements in quality of life or major adverse cardiovascular events (16). An expert consensus in China stated that CCTA is suitable for patients with moderate or low risk, those with atypical chest pain and clinically suspected CAD but without definitive diagnosis by ECG, and those who cannot tolerate other imaging options (17), but the study did not examine the value for the other patient populations. Another expert consensus in China highlights the promising outcomes of CT-FFR but also that there is a lack of standardized data interpretation (18).

A knowledge, attitude, and practice (KAP) survey is a structured approach widely employed in sociological and psychological research and has increasingly found utility in the medical domain (19). Such studies assess the KAP within a specific target population, most often in the context of public health or behavioral research. These studies are designed to systematically evaluate what people know (knowledge), how people feel or believe (attitudes), and what people actually do (practices). KAP surveys primarily use structured or semi-structured questionnaires, which may be self-administered or completed via interviews (20,21,22). Most KAP studies are quantitative, focusing on collecting and analyzing numerical data to measure the prevalence of knowledge, attitudes, or behaviors within a population (20,21,22). KAP studies help pinpoint what people know, misunderstand, or are unaware of regarding a specific topic. By highlighting gaps in knowledge or misconceptions, these surveys guide the creation of focused educational or intervention programs. Understanding how people feel about an issue (whether they support, oppose, or are indifferent) helps in predicting behavior change likelihood and in designing culturally sensitive policies and campaigns. These studies uncover real-world behaviors rather than assumptions, providing a factual basis for strategic planning (for example, measuring adherence to health guidelines, vaccination uptake, or hygiene practices). Because KAP studies reveal both strengths and weaknesses in population knowledge and behavior, interventions can be more precisely tailored, increasing efficiency and impact (20,21,22).

Of particular interest is its application to assess the KAP level of physicians regarding CT-FFR technology. As the main decision-makers and practitioners in the medical team, doctors have professional knowledge and clinical experience, and their recognition and acceptance directly affect the promotion and application of new technologies, as well as patients’ acceptance of new technologies and their application in clinical practice. Technologists and nurses are important in cardiac care, as they are involved in collaborative treatment teams, knowledge dissemination and training, patient education and support, teamwork, and the exchange of opinions. As medical technicians’ awareness, attitudes, and practice behaviors may exert a profound influence on the adoption of innovative technologies, conducting a KAP study can yield invaluable insights into the current state of medical technicians’ understanding, perceptions, and utilization of CT-FFR in the context of diagnosing coronary artery disease. Moreover, this survey can effectively pinpoint potential challenges, thereby assisting in the optimization of health education and disease management strategies.

Thus, this study aimed to determine the KAP of cardiothoracic physicians working in cardiology and radiology departments toward the CT-FFR technology, including their understanding of the underlying principles and scope of application. The results could help determine the areas that would warrant improvements in the future.

## Methods

### Study design and participants

This cross-sectional study was conducted in hospitals located in Beijing, Hebei, Shandong, and Guangdong regions from May to June 2023, with a focus on cardiothoracic physicians in cardiology and radiology as the primary study participants. The sample size was calculated using the Fisher et al. (23,24) formula: n = Z^2^ × P × (1-P)/D^2^, where ‘n’ is the sample size, ‘Z’ is the Z-score corresponding to the desired confidence level (e.g., 1.96 for 95%), ‘P’ is the estimated prevalence of the event of interest (P = 0.5 was used here), and ‘D’ is the desired margin of error according to multiple sources (D = 0.072 here). Hence, the minimal sample size in this study was n = 196.

Inclusion criteria for participation were limited to cardiothoracic physicians in cardiology and radiology, while those with incomplete questionnaire responses were excluded. The distribution of questionnaires took place through WeChat groups utilizing the “Questionnaire Star” platform.

This study was approved by the Ethics Committee of Tianjin Medical University General Hospital (IRB2023-YX-173-01). All the participants who constituted the survey population provided written informed consent.

### Procedures

The questionnaire design for this study was informed by previous publications (11,12,13,14,16,17) and the “Chinese Expert Consensus on the Clinical Application of Coronary CT Fractional Flow Reserve” (18). Following the initial design, feedback was obtained from four experts, including two cardiovascular specialists (one associate chief physician and one chief physician) and two radiology specialists (both chief physicians). The questionnaire underwent revisions based on their valuable suggestions and subsequently underwent a small-scale pilot test. The reliability of the questionnaire was assessed, yielding a satisfactory Cronbach’s α coefficient of 0.884. The Kaiser-Meyer-Olkin (KMO) value was 0.828.

The final version of the questionnaire was developed in Chinese and comprised three dimensions:

1. demographic information, consisting of 10 questions;
2. attitude dimension, encompassing 15 questions, all rated on a five-point Likert scale ranging from 1 to 5 to gauge the participants’ attitudes, yielding a total score range of 15 to 75;
3. The willingness dimension, comprising eight questions, was also rated on a five-point Likert scale from 1 to 5 to assess the level of willingness, resulting in a total score range of 8 to 40.

The median of each score was used as the cutoff for determining poor or good KAP. The median is a statistically defensible and pragmatic cutoff in KAP studies, particularly useful when there is no widely accepted cutoff for “adequate” KAP, the score distribution is non-normal or contains outliers, and researchers need a context-specific threshold to categorize respondents for further analysis or intervention (25).

In order to facilitate data collection, an online questionnaire was created using the WeChat-based Questionnaire Star mini-program, and a QR code was generated so that participants could access and complete the questionnaire via WeChat. In order to ensure data quality and completeness, each IP address was restricted to one submission, and all items in the questionnaire were made mandatory. Data collected through the platform were exported to an Excel spreadsheet for further analysis. The research team members meticulously reviewed the questionnaires to ensure the integrity, internal coherence, and reasonability of the data.

### Statistical analysis

Quantitative variables were presented as Mean ± SD. Intergroup comparisons for normally distributed data were conducted using the t-test or ANOVA, while the Mann-Whitney test or Kruskal-Wallis H test was used for non-normally distributed data. The attitude and willingness scores were not normally distributed, and the median was used as the cutoff point. Categorical variables were described in terms of frequency (percentage). The correlations between attitude and practice scores were examined using Spearman’s analysis. Univariable and multivariable logistic regression analyses were performed. Statistical significance was defined as a P-value less than 0.05. All statistical analyses were carried out using SPSS 26.0 software (IBM, Armonk, NY, USA).

## Results

### Demographic characteristics

There were 275 questionnaires, but eight were excluded because the answer time was too short (<114 s) or too long (>1800 s), and two did not agree with their data being used for research, leaving 265 questionnaires in the analysis. The 265 cardiothoracic physicians in cardiology and radiology departments consisted of 151 males (57.0%) and 114 females (43.0%). Among the age groups, the highest representation was observed in the 31–40 years range, accounting for 45.3% of the participants, while those above 50 years were the smallest subgroup at 9.4%. A significant proportion of the physicians held a graduate degree (61.1%), followed by a bachelor’s degree (35.1%). The majority of participants worked as physicians (91.3%). Moreover, a considerable number of physicians had over ten years of work experience (54.7%) **(Table S1)**.

### Attitude and willingness

**Figure 1:**
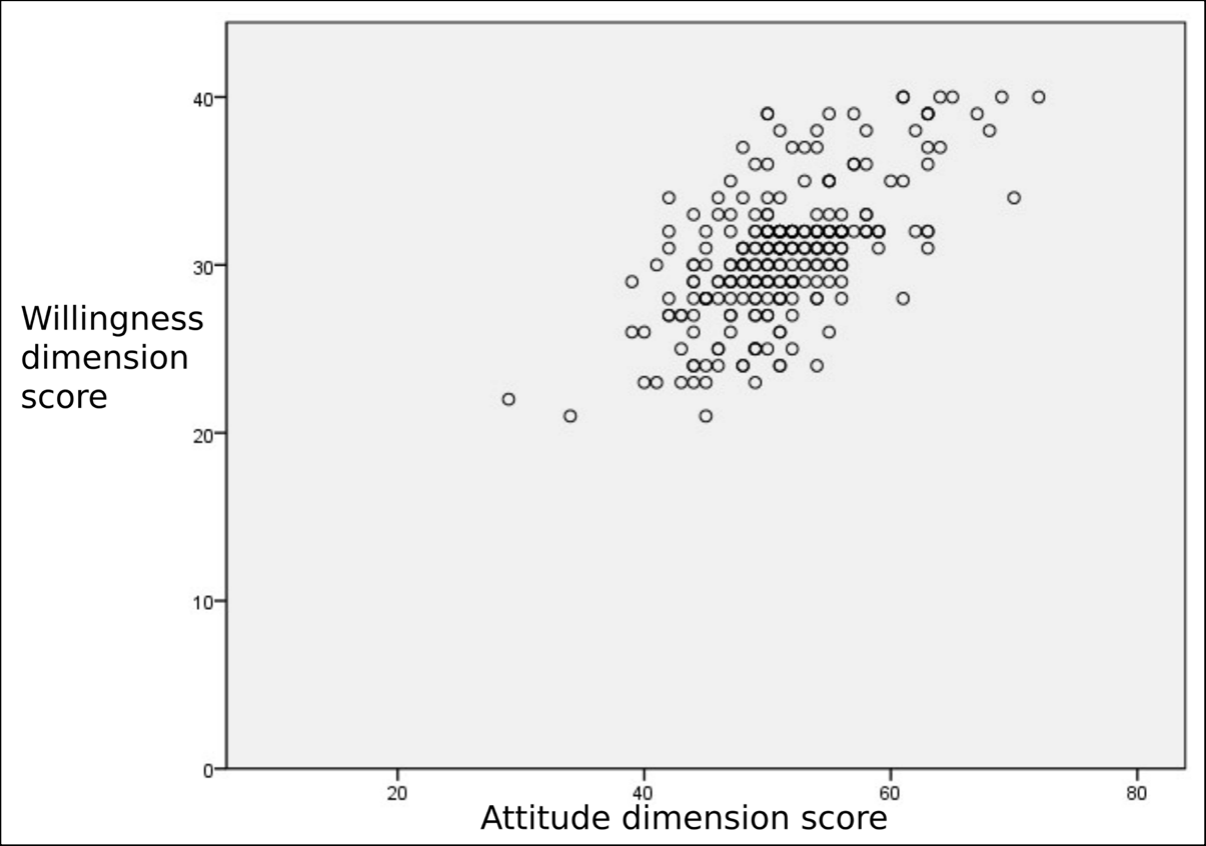
Scatter plot of willingness and attitude dimension scores.

The correlation coefficient between the attitude and the willingness dimension was found to be 0.571 (p < 0.001), indicating a moderate positive correlation (Figure 1).

Many respondents reported being very familiar or familiar with CT-FFR (43.8%). Irrespective of familiarity, the respondents believed CR-FFR could avoid unnecessary invasive procedures (64.5%) and assess the entire coronary artery’s blood flow reserve (60.8%). Furthermore, about two-thirds agreed that CT-FFR could be helpful for post-treatment evaluation of coronary heart disease (67.9%), and two-fifths believed that its clinical application value could exceed that of invasive wire-based FFR (40.8%). However, some concerns were evident, especially about accuracy (38.1%) and potential for medical litigation (42.3%) (Tables 1 and **S2**).

**Table 1:** Distribution of Attitude dimension responses.


	**a) Very Familiar (5 points)**	**b) Familiar**	**c) Average**	**d) Not Familiar**	**e) Completely Unfamiliar (1 Point)**

**1. How familiar are you with CT-derived fractional flow reserve (CT-FFR) technology?**	25 (9.5)	91 (34.3)	97 (36.6)	42 (15.8)	10 (3.8)

	**a) Strongly Agree (5 points)**	**b) Agree**	**c) Neutral**	**d) Disagree**	**e) Completely Disagree (1 point)**

**2. Do you think conventional invasive wire based FFR increases procedural steps, time and cost, limiting its widespread us in China?**	32 (12.1)	137 (51.6)	85 (32.1)	11 (4.2)	0 (0.0)

	**a) Strongly Agree (5 points)**	**b) Agree**	**c) Neutral**	**d) Disagree**	**e) Completely Disagree (1 point)**

**3. Do you believe CT-FFR can avoid unnecessary invasive procedures and related complications?**	32 (12.1)	171 (64.5)	53 (20.0)	9 (3.4)	0 (0.0)

	**a) Strongly Agree (5 points)**	**b) Agree**	**c) Neutral**	**d) Disagree**	**e) Completely Disagree (1 point)**

**4. Do you think CT-FFR can assess the entire coronary artery’s blood flow reserve, compensating for the limitation of invasive wire-based FFR, which can only evaluate individual vessel stenosis?**	35 (13.2)	161 (60.8)	63 (23.8)	6 (2.2)	0 (0.0)

	**a) Strongly Agree (5 points)**	**b) Agree**	**c) Neutral**	**d) Disagree**	**e) Completely Disagree (1 point)**

**5. Do you believe CT-FFR technology can be used for post-treatment evaluation of coronary heart disease to obtain more prognostic information?**	36 (13.6)	180 (67.9)	45 (17.0)	4 (1.5)	0 (0.0)

	**a) Very High (5 Points)**	**b) High**	**c) Average**	**d) Low**	**e) Very Low (1 Point)**

**6. How do you rate the acceptance of CT-FFR technology among coronary heart disease patients?**	25 (9.4)	125 (47.2)	97 (36.6)	17 (6.4)	1 (0.4)

	**a) Very Worried (1 Point)**	**b) Worried**	**c) Neutral**	**d) Not Worried**	**e) Not Worried at all (5 Points)**

**7. Compared to conventional invasive wire-based FFR results, are you concerned about the accuracy of CT-FFR results?**	15 (5.7)	113 (42.6)	101 (38.1)	34 (12.8)	2 (0.8)

	**a) Very Worried (1 Point)**	**b) Worried**	**c) Neutral**	**d) Not Worried**	**e) Not Worried at all (5 Points)**

**8. Are you concerned that inaccurate CT-FFR results may lead to subsequent medical litigation?**	15 (5.6)	85 (32.1)	112 (42.3)	53 (20.0)	0 (0.0)

	**a) Strongly Agree (5 points)**	**b) Agree**	**c) Neutral**	**d) Disagree**	**e) Completely Disagree (1 point)**

**9. Do you think the popularization of CT-FFR technology will reduce the time and cost of diagnosis and treatment?**	18 (6.8)	147 (55.5)	74 (27.9)	26 (9.8)	0 (0.0)

	**a) Very Helpful (5 Points)**	**b) Somewhat Helpful**	**c) Possibly Helpful**	**d) Not Very Helpful**	**e) Not Helpful at All (1 Points)**

**10. In your practice, how helpful do you think CT-FFR technology is for the diagnosis of coronary heart disease?**	57 (21.5)	159 (60.0)	45 (17.0)	3 (1.1)	1 (0.4)

	**a) Strongly Agree (5 points)**	**b) Agree**	**c) Neutral**	**d) Disagree**	**e) Completely Disagree (1 point)**

**11. Do you believe the clinical application value of CT-FFR technology can exceed that of invasive wire-based FFR?**	12 (4.5)	87 (32.8)	108 (40.8)	53 (20.0)	5 (1.9)

	**a) Strongly Agree (5 points)**	**b) Agree**	**c) Neutral**	**d) Disagree**	**e) Completely Disagree (1 point)**

**12. Do you think CT-FFR technology has greater development potential?**	45 (17.0)	165 (62.3)	52 (19.6)	3 (1.1)	0 (0.0)

	**a) Very High (5 Points)**	**b) High**	**c) Average**	**d) Low**	**e) Very Low (1 Point)**

**13. How do you rate the promotion of CT-FFR technology in your hospital?**	11 (4.2)	61 (23.0)	110 (41.5)	51 (19.2)	32 (12.1)

	**a) Very Necessary (5 Points)**	**b) Necessary**	**c) Neutral**	**d) Not Very Necessary**	**e) Completely Unnecessary (1 Point)**

**14. Do you think it is necessary to further promote the application of CT-FFR technology?**	36 (13.6)	171 (64.5)	55 (20.8)	3 (1.1)	0 (0.0)

	**a) Strongly Agree (5 points)**	**b) Agree**	**c) Neutral**	**d) Disagree**	**e) Completely Disagree (1 point)**

**15. Do you think it will take a considerable amount of time for the widespread adoption of CT-derived fractional flow reserve (CT-FFR) technology?**	23 (8.7)	157 (59.2)	70 (26.4)	15 (5.7)	0 (0.0)


About half of the participants strongly agreed or agreed (53.2%) that CT-FFR should become the standard method for diagnosing coronary artery disease. Moreover, 71.6% expressed willingness to try and apply CT-FFR technology if they had not used it before. Regarding patient education, 70.6% were willing to introduce the differences between CT-FFR and invasive examinations to the patients. About two-thirds of the participants expressed willingness to actively learn about the technical principles and clinical applications of CT-FFR and to receive further training and education on CT-FFR (Table 2).

**Table 2:** Distribution of attitude dimension responses.


	**a) Strongly Agree (5 points)**	**b) Agree**	**c) Neutral**	**d) Disagree**	**e) Completely Disagree (1 point)**

**1. Do you think CT-derived fractional flow reserve (CT-FFR) technology should become the standard method for diagnosing coronary artery disease?**	17 (6.4)	124 (46.8)	101 (38.1)	23 (8.7)	0 (0.0)

	**a) Very Willing (5 points)**	**b) Willing**	**c) Average**	**d) Unwilling**	**e) Completely Unwilling (1 Point)**

**2. If you have not used CT-derived fractional flow reserve (CT-FFR) technology, would you be willing to try and apply this technology?**	37 (14.0)	190 (71.6)	36 (13.6)	2 (0.8)	0 (0.0)

	**a) Very Willing (5 points)**	**b) Willing**	**c) Average**	**d) Unwilling**	**e) Completely Unwilling (1 Point)**

**3. Are you willing to introduce the differences between CT-derived fractional flow reserve (CT-FFR) technology and invasive wire-based FFR technology to patients?**	35 (13.1)	187 (70.6)	41 (15.5)	2 (0.8)	0 (0.0)

	**a) Strongly Recommend (5 Points)**	**b) Recommend**	**c) Might Recommend**	**d) Not Recommend**	**e) Completely not recommend (1 Point)**

**4. Would you recommend the use of CT-derived fractional flow reserve (CT-FFR) technology for coronary functional assessment to patients?**	27 (10.2)	149 (56.2)	86 (32.5)	3 (1.1)	0 (0.0)

	**a) Very Willing (5 points)**	**b) Willing**	**c) Average**	**d) Unwilling**	**e) Completely Unwilling (1 Point)**

**5. Are you willing to actively learn about the technical principles, workflow, precautions, and clinical applications of CT-derived fractional flow reserve (CT-FFR)?**	56 (21.1)	173 (65.3)	36 (13.6)	0 (0.0)	0 (0.0)

	**a) Very Willing (5 points)**	**b) Willing**	**c) Average**	**d) Unwilling**	**e) Completely Unwilling (1 Point)**

**6. Are you willing to receive further training and education on CT-derived fractional flow reserve (CT-FFR) technology?**	58 (21.9)	177 (66.8)	30 (11.3)	0 (0.0)	0 (0.0)

	**a) Considered deeply multiple times (5 points)**	**b) Considered**	**c) Occasionally thought about**	**d) No Considered**	**e) Never paid attention (1 point)**

**7. Have you considered the need for further improvement in the application of CT-derived fractional flow reserve (CT-FFR) technology and what efforts are needed?**	13 (4.9)	131 (49.4)	73 (27.5)	38 (14.3)	10 (3.9)

	**a) Very Willing (5 points)**	**b) Willing**	**c) Average**	**d) Unwilling**	**e) Completely Unwilling (1 Point)**

**8. Are you willing to participate in clinical research using CT-derived fractional flow reserve (CT-FFR) technology?**	48 (18.1)	167 (63.0)	45 (17.0)	5 (1.9)	0 (0.0)


### Correlation analysis

**Figure 2:**
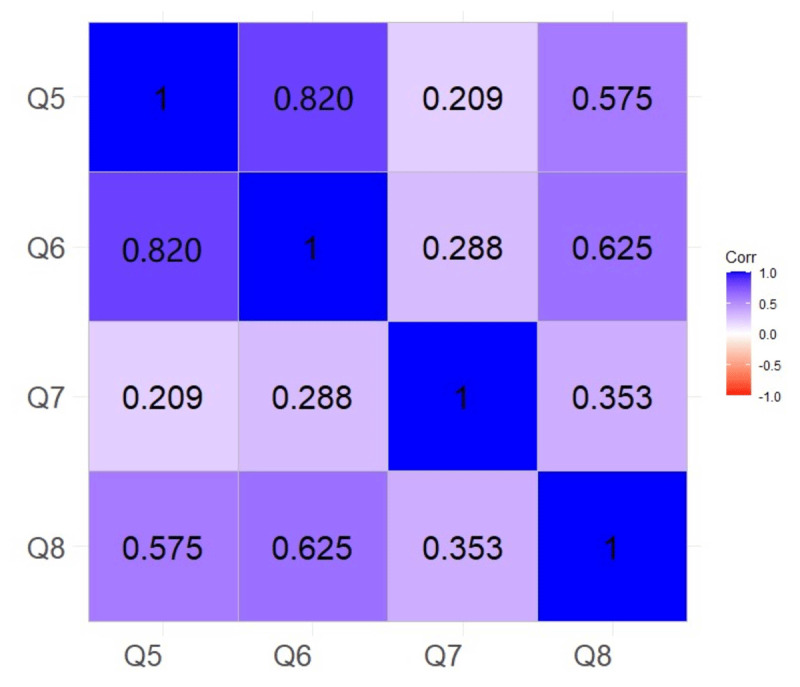
Willingness dimension part topic relevance.

The analysis revealed a strong positive association (r = 0.820, p < 0.001) between participants’ willingness to actively learn about CT-FFR technology (Q5) and their openness to receive further training (Q6). Additionally, participants’ willingness to actively learn (Q5) exhibited a moderate positive correlation with their interest in participating in clinical research involving CT-FFR (Q8) (r = 0.575, p < 0.001) (Figure 2).

**Figure 3:**
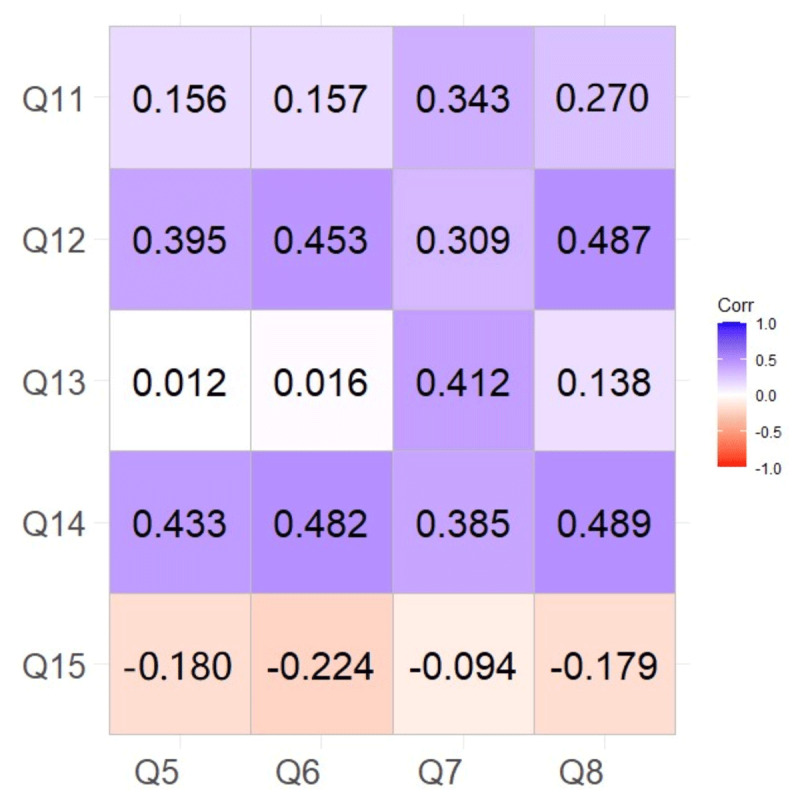
Correlation between the question of attitude and the willingness dimension.

Participants’ belief in the greater development potential of CT-FFR technology (Q12) showed strong positive correlations with their willingness to actively learn about CT-FFR (Q5) (r = 0.395, p < 0.001), their willingness to receive further training (Q6) (r = 0.453, p < 0.001), their consideration of the need for improvement in CT-FFR application (Q7) (r = 0.309, p < 0.001), and their willingness to participate in clinical research involving CT-FFR (Q8) (r = 0.487, p < 0.001). Similarly, participants’ perception of the necessity for further promotion of CT-FFR application (Q14) exhibited significant positive correlations with their willingness to actively learn (Q5) (r = 0.433, p < 0.001), their willingness to receive further training (Q6) (r = 0.482, p < 0.001), their consideration of the need for improvement (Q7) (r = 0.385, p < 0.001), and their willingness to participate in clinical research (Q8) (r = 0.489, p < 0.001). However, the rating of promotion of CT-FFR technology in the hospital (Q13) did not show significant correlations with the Willingness Dimension items (Figure 3).

### Logistic regression

Among these factors, physicians were found to be less likely to have positive attitudes (OR = 0.312, 95% CI: 0.112–0.867, p = 0.026) compared with other occupations. Similarly, participants working in hospitals specialized in cardiovascular diseases showed a higher likelihood of positive attitudes (OR = 3.085, 95% CI: 1.224–7.771, p = 0.017) when compared to those in other types of medical institutions. After adjusting for potential confounding variables in the multivariable analysis, physicians (OR = 0.343, 95% CI: 0.123–0.957, p = 0.041) and participants in specialist hospitals (OR = 0.354, 95% CI: 0.140–0.896, p = 0.028) maintained significant associations with negative attitudes. The logistic regression analysis of participants’ willingness (categorized based on the cutoff value of ≥31 and <31) showed that a high attitude dimension score (OR = 6.280, 95%CI: 3.673–10.736, p < 0.001) was independently associated with willingness **(Table S3)**.

## Discussion

The findings of this study provide valuable insights into the perceptions and attitudes of cardiothoracic physicians in cardiology and radiology toward CT-FFR technology, as well as their willingness to adopt and use this novel diagnostic method for coronary artery disease.

### Attitudes toward CT-FFR technology

The physician community shows a cautious optimism about CT-FFR, but there is a huge gap between this positive willingness and its practical application due to the multiple real obstacles like financial resources, workload, time, and access to the CT-FFR technology. In addition, concerns about the accuracy of CT-FFR can be related to motion artifacts, severe calcifications, and microvascular disease that may lead to differences between CT-FFR and invasive FFR (26). A significant number of participants displayed familiarity with CT-FFR and expressed confidence in its ability to reduce the necessity for invasive procedures while providing a comprehensive evaluation of coronary artery blood flow reserve. These attitudes are encouraging for the potential seamless integration of CT-FFR into clinical practice. The majority of participants held favorable views toward CT-FFR, with a notable percentage acknowledging its capacity to improve patient care by reducing the need for invasive procedures and facilitating a comprehensive assessment of coronary artery health. This cautious reception could be attributed to CT-FFR’s promising diagnostic performance and its potential to advance patient care in the field of cardiology (27,28,29).

### Concerns among physicians toward CT-FFR technology

However, the study also sheds light on certain reservations among participants, particularly regarding the accuracy of CT-FFR results compared with invasive wire-based FFR. A notable proportion of participants expressed concerns about the potential risk of medical litigation arising from inaccurate CT-FFR results. Physicians are the ones signing the reports and bearing responsibility for misdiagnosis or inaccurate results. Physicians’ negative attitudes toward CT-FFR may be due to factors such as their professional awareness, sense of responsibility, education, industry culture, and traditional preferences. The lack of standardized interpretation is a major limitation of CT-FFR, which is recognized by the Chinese expert consensus on CT-FFR (18). These apprehensions signify that despite some positive attitudes toward CT-FFR, there exist lingering reservations and uncertainties regarding the technology’s reliability and potential legal implications. While this technique generally exhibits high diagnostic accuracy, specific cases of severe calcifications, poor imaging quality, or microvascular disease may lead to diagnostic inconsistencies. If not properly managed, these challenges can pose a risk of misdiagnosis or overtreatment. More education and training may be needed to promote physician acceptance of this new technology. In order to alleviate these concerns, studies should be conducted to standardize data interpretation protocols, improve algorithm robustness, enhance physician training, and enable liability insurance coverage as viable solutions. Additional studies demonstrating (or not) the effectiveness, safety, and healthcare economics of the technology are needed to reach an informed decision about its widespread use in the clinical setting. These measures would be designed to reduce the likelihood of controversy while increasing confidence in the clinical application of the technology. Consequently, it is imperative for the medical community and manufacturers to address these concerns diligently through persistent research efforts, validation studies, and transparent communication, elucidating the precision and safety of the technology. Manufacturers’ enthusiasm will have to be carefully balanced against a proper academic approach to ensure maximum profits for the patients and not for the industry.

### Diagnostic accuracy of the CT-FFR technology

Previous studies have explored the impact of vessel calcification on the diagnostic accuracy of CT-FFR (29,30,31), further contributing to the understanding and refinement of this novel approach. Indeed, Tesche et al. (29) highlighted the potential for CT-FFR as a single examination that could guide patient management strategies. In addition, Tang et al. (30) showed that CT-FFR performed well in detecting lesion-specific ischemia. Tesche et al. (31) showed that the diagnostic performance of CT-FFR was related to the artery calcium scores. While CT-FFR has proven valuable in diagnosing and treating CAD, certain limitations persist. Clinical evidence derived from CT-FFR is contingent on specific software, cautioning against broad generalizations across different software products. In addition, CT-FFR demands high-quality CCTA images, leaving a substantial proportion of images unevaluable. The diagnostic efficacy of CT-FFR for severely calcified lesions remains uncertain, and a notable gap exists in clinical evidence supporting its application post-percutaneous coronary intervention or coronary artery bypass grafting. The presence of such concerns, specifically regarding the accuracy of CT-FFR results compared with invasive wire-based FFR and the potential for medical litigation, may impede the widespread implementation of CT-FFR (32,33,34).

### Willingness to use CT-FFR technology

While the participants exhibited cautious attitudes, it is crucial to recognize the salience of accuracy-related concerns and the necessity of enhancing legal clarity to reach a better understanding of the pros and cons balance of CT-FFR technology. Regarding physicians’ inclination to acquire knowledge about CT-FFR, the findings unveiled that a majority of participants demonstrated receptiveness to CT-FFR. A majority of physicians displayed a willingness to explore and potentially apply CT-FFR technology, educate their patients about its advantages, and engage in enhancing their comprehension and proficiency in the technology. Furthermore, many indicated an eagerness to partake in clinical research endeavors involving CT-FFR. The study’s findings underscore a notable willingness among physicians to enhance their comprehension of CT-FFR technology, indicative of receptiveness toward integrating this innovative approach into their clinical practice. Furthermore, mounting evidence supports the utility of CT-FFR in guiding revascularization decisions, and the diagnostic performance of machine learning-based CT-FFR is anticipated to improve with advancements in image quality (10,11,12,13,14).

### Cost-effectiveness of the CT-FFR technology

CT-FFR is an advanced noninvasive method to assess the physiological significance of coronary lesions. Despite its technical promise and guideline recommendations, its widespread clinical adoption remains slow, with one of the most formidable and systematic barriers being the lack of robust, generalizable cost-effectiveness data and unclear prospects for medical insurance reimbursement. Physicians are less likely to recommend or integrate a technology whose incremental clinical benefit versus cost is not convincingly established in the local healthcare context (35,36). Mixed or preliminary cost-effectiveness evidence—such as data limited to certain populations or derived from short-term outcomes—feeds clinician uncertainty, especially when compared with well-established, reimbursed alternatives (e.g., stress tests, invasive angiography) (36,37). When coverage or out-of-pocket costs are ambiguous, clinicians may avoid CT-FFR to shield patients from unexpected expenses and themselves from enterprising into nonstandard care pathways. This is particularly true since procedural and diagnostic costs can be substantial and variable (38). The financial policies of technology use trickle down to everyday practice. In settings with partial or delayed reimbursement trends—such as when some, but not all, payers or regions cover CT-FFR—clinical behaviors become inconsistent (37,38). A lack of clear insurance endorsement means physicians cannot reliably predict patient access or institutional budget impact, further muting enthusiasm for broad adoption. The uncertainty around whether CT-FFR will be supported by insurance strongly correlates with lower stated intentions to use or trial the modality, even among clinicians otherwise convinced by the technology’s clinical promise. This is a classic finding in implementation science, and recent logistic regression analyses reinforce that reimbursement concerns are among the strongest predictors—often outweighing concerns about technical validity or workflow changes. Without clear cost-effectiveness, even hospital-level champions for innovation find it hard to justify the investment, as administrative and financial committees demand evidence of downstream savings or improved outcomes (35,36,39). Often, tertiary or quaternary centers with greater research focus and more flexible funding streams, specialist hospitals might pilot CT-FFR in select patient groups despite reimbursement uncertainty. However, even here, wider roll-out is often limited to research protocols or select populations as administrators are cautious to assume costs not guaranteed to be recouped (37). These settings, especially in resource-constrained or rural regions, are more sensitive to reimbursement clarity. They typically have less financial buffer for innovation risk and must prioritize technologies with clear, reimbursable pathways. Consequently, adoption of CT-FFR in these environments is highly restricted until reimbursement is formalized and/or local cost-savings are demonstrated in pragmatic terms, beyond trial settings (37,40).

### Correlations between attitudes and willingness

The correlation analysis between the attitude and willingness dimensions revealed a moderate positive correlation, indicating that physicians with higher attitude scores toward CT-FFR technology were also more inclined to engage in learning about it, seek further training, and participate in clinical research involving CT-FFR. This finding emphasizes the importance of addressing attitude-related factors when promoting the integration of novel technologies in clinical practice (41,42). Comprehensive strategies need to be implemented to cultivate a proper attitude (i.e., balancing pros and cons) toward CT-FFR among cardiothoracic physicians in cardiology and radiology. Targeted educational programs could provide in-depth insights into the benefits and applications of CT-FFR, addressing any misconceptions or uncertainties. Interactive workshops could further facilitate hands-on experience and open dialogues, fostering a collaborative learning environment for physicians to share experiences and best practices. In addition, incorporating case studies that highlight successful outcomes with CT-FFR may serve to alleviate any skepticism and build confidence in its effectiveness. Furthermore, establishing ongoing communication channels and forums for continuous learning and feedback could ensure that physicians stay abreast of the latest developments about CT-FFR. Moreover, the correlation analysis demonstrated interesting associations among participants’ responses, indicating that higher attitude scores toward CT-FFR are closely linked to an increased willingness to learn, receive additional training, consider improvements, and engage in clinical research (43). This interdependence highlights the importance of fostering positive attitudes to stimulate active engagement with CT-FFR technology. Of course, attitudes and willingness are dynamic processes that can change, either positively or negatively, with accumulating data about new technology. Nevertheless, the findings emphasize the pivotal role of positive attitudes in influencing various aspects of willingness, reinforcing the need to cultivate proper perceptions of new technology. In China, the hospital classification system differs from that in Western countries; however, public tertiary hospitals in China are generally comparable to university medical centers or comprehensive medical centers in other countries. These hospitals play an important role in healthcare, providing high-level medical care, teaching, and research. The logistic regression analysis revealed that both physician status and employment in specialist hospitals were significantly associated with higher attitude scores toward CT-FFR, underscoring the potential influence of institutional and professional factors on technology acceptance (44). Furthermore, the study highlighted the critical role of attitude in motivating physicians’ willingness to embrace CT-derived FFR, emphasizing the need for interventions that enhance their understanding and appreciation of this innovative diagnostic method. These insights provide a valuable foundation for targeted strategies to enhance clinical practice, ultimately improving diagnostic accuracy and patient care. However, in contrast to expectations, the study did not observe statistically significant associations between demographic variables such as gender, age, and education level and the KAP scores toward CT-FFR.

### Other technologies for hemodynamics

Strategies other than invasive angiography and noninvasive CCTA/CT-FFR exist to evaluate hemodynamics in CAD, including cardiac magnetic resonance imaging (CMR), radionuclide myocardial perfusion imaging, and stress echocardiography (17). Still, CT systems are usually more widely available than CMR systems; the examination takes less time and is less susceptible to motion artifacts, and the images have a higher resolution (17). CCTA does not involve radioactive isotopes that can have availability issues and pose certain safety risks. Echocardiography is operator-dependent, and accuracy or results can be variable among operators (45). Hence, CT-FFR appears promising in avoiding the limitations of the other modalities. Still, the availability of machines certified for CT-FFR can vary among countries and provinces, possibly affecting the willingness of the physicians to use them.

### Integration of the CT-FFR technology in the clinical setting

A tiered training approach is recommended to improve the integration and effective use of CT-FFR technology among physicians. This approach should be tailored to the different levels of expertise and experience. For junior physicians and trainees, fundamental courses covering the basics of CT-FFR technology, including its principles, benefits, and limitations, should be implemented. These courses should emphasize hands-on training with simulation tools to build initial confidence. For mid-level physicians, intermediate training sessions focusing on case studies, interpretation of results, and integration of CT-FFR data into clinical decision-making are essential. Advanced workshops for senior physicians should delve into complex cases, troubleshooting, and the latest research findings.

In addition to these tiered training programs, regular interdisciplinary seminars should be organized to foster collaboration between cardiologists and radiologists. These seminars should highlight the clinical scenarios where CT-FFR is the most beneficial, discuss the accuracy and limitations of the technology, and review the current guidelines and best practices. Moreover, continuous professional development should include updates on the latest advancements in CT-FFR and feedback sessions to address any persistent challenges or concerns. Establishing a mentorship program where experienced users of CT-FFR guide less experienced colleagues can also enhance practical understanding and application.

Finally, transparent communication regarding the accuracy and limitations of CT-FFR, particularly concerning its use in specific clinical situations, should be emphasized. Clear guidelines on the interpretation of borderline CT-FFR values and their implications for clinical management should be developed to ensure consistent and accurate use.

### Future perspectives

While CT-FFR has potential clinical benefits in CAD diagnosis (7,8), its widespread adoption and application in clinical practice face challenges. CT-FFR is not currently included in practice guidelines in China despite available data that CT-FFR may improve decision-making, patient management, and resource utilization (10,11,12,13,14,15). Still, there are some concerns about healthcare economics and the lack of standardized interpretation (16,17,18). Hence, performing additional studies and organizing expert opinion committees are essential to delineate the role of CT-FFR in the management of CAD and ultimately incorporate it in guidelines to guide its judicious use.

### Limitations

This cross-sectional study examining the attitudes and willingness of cardiothoracic physicians in cardiology and radiology in China toward using CT-FFR as a diagnostic tool for CAD has several limitations. Firstly, the cross-sectional design restricts the establishment of causal relationships and temporal changes. Secondly, all participants were from a limited geographical area, limiting the generalizability of the results. Although some KAP studies are sometimes performed at the regional or national level, most KAP studies are performed locally because the differences in healthcare resources, healthcare policies, and training among regions can introduce variability in the results. Thirdly, potential biases, including sampling bias and social desirability bias (46,47), may influence the validity of the results. The use of WeChat for sampling and questionnaire completion can lead to sampling and selection bias by excluding those with lower digital aptitudes and skewing inclusion toward those who are more active on social media. Fourthly, the questionnaire did not contain items about operational issues, and the processing time and workflow of CT-FFR were not considered in the present study and might affect the willingness of the physicians toward it, especially in the emergency setting. This study was a survey of the participants’ attitudes and willingness toward CCTA and CT-FFR, and the lack of knowledge and experience level of the surveyed population on CCTA and CT-FFR probably affected the results of this study to a certain extent. Nevertheless, the lack of knowledge of the population can also be further reflected in the attitude and willingness scores. Therefore, it is necessary to strengthen the publicity and education of CCTA and CT-FFR content and improve their recognition.

## Conclusions

This study highlights the promising acceptance and cautious attitudes toward CT-FFR technology among cardiothoracic physicians in cardiology and radiology, with a majority expressing willingness to explore and implement it in their clinical practice, filling a gap in the literature. However, concerns about accuracy and potential legal ramifications should be addressed to enhance confidence in CT-FFR. By understanding the factors influencing attitudes and willingness, healthcare organizations and policymakers can design effective strategies to foster the integration of CT-FFR into routine clinical decision-making. A broader use of CT-FFR could translate into better patient outcomes through less invasive examinations, lower risk, and optimization of CAD diagnosis and management. Further research is warranted to assess long-term outcomes, cost-effectiveness, and patient-related factors that might impact the widespread adoption of CT-FFR in real-world clinical settings.

## Data Accessibility Statement

All data generated or analyzed during this study are included in this article.

## Additional File

The additional file for this article can be found as follows:

10.5334/gh.1477.s1 Supplementary File. Tables S1 to S3.

## References

[1] Kim KJ, Choi SI, Lee MS, Kim JA, Chun EJ, Jeon CH. The prevalence and characteristics of coronary atherosclerosis in asymptomatic subjects classified as low risk based on traditional risk stratification algorithm: Assessment with coronary CT angiography. Heart. 2013;99(15):1113–1117. DOI: 10.1136/heartjnl-2013-303631 23723445

[2] Han P, Tang J, Wang X, Su Y, Li G, Deng K. Research on the distribution spectrum of atherosclerotic plaques in patients with suspected coronary artery disease and the noninvasive screening model for coronary atherosclerosis burden. Quant Imaging Med Surg. 2021;11(7):3274–3285. DOI: 10.21037/qims-20-901 34249653 PMC8250030

[3] Holman RR, Coleman RL, Chan JCN, Chiasson J-L, Feng H, Ge J, et al. Effects of acarbose on cardiovascular and diabetes outcomes in patients with coronary heart disease and impaired glucose tolerance (ACE): A randomised, double-blind, placebo-controlled trial. Lancet Diabetes Endocrinol. 2017;5(11):877–886. DOI: 10.1016/S2213-8587(17)30309-1 28917545

[4] Cheng M, Cheng M, Wei Q. Association of myeloperoxidase, homocysteine and high-sensitivity C-reactive protein with the severity of coronary artery disease and their diagnostic and prognostic value. Exp Ther Med. 2020;20(2):1532–1540. DOI: 10.3892/etm.2020.8817 32765675 PMC7388560

[5] Gao H, Liu S, Cai H, Chen D, Fu X, Zhao S, et al. Guipi decoction for coronary heart disease: A protocol for a systematic review and meta-analysis. Medicine (Baltimore). 2020;99(32):e21589. DOI: 10.1097/MD.0000000000021589 32769912 PMC7593082

[6] Han C, Peng Y, Yang X, Guo Z, Yang X, Su P, et al. Declined plasma microfibrillar-associated protein 4 levels in acute coronary syndrome. Eur J Med Res. 2023;28(1):32. DOI: 10.1186/s40001-023-01002-z 36650606 PMC9847181

[7] Investigators S-H, Newby DE, Adamson PD, Berry C, Boon NA, Dweck MR, et al. Coronary CT angiography and 5-year risk of myocardial infarction. N Engl J Med. 2018;379(10):924–933. DOI: 10.1056/NEJMoa1805971 30145934

[8] Min JK, Leipsic J, Pencina MJ, Berman DS, Koo BK, van Mieghem C, et al. Diagnostic accuracy of fractional flow reserve from anatomic CT angiography. JAMA. 2012;308(12):1237–1245. DOI: 10.1001/2012.jama.11274 22922562 PMC4281479

[9] Lossnitzer D, Klenantz S, Andre F, Goerich J, Schoepf UJ, Pazzo KL, et al. Stable patients with suspected myocardial ischemia: comparison of machine-learning computed tomography-based fractional flow reserve and stress perfusion cardiovascular magnetic resonance imaging to detect myocardial ischemia. BMC Cardiovasc Disord. 2022;22(1):34. DOI: 10.1186/s12872-022-02467-2 35120459 PMC8817462

[10] Douglas PS, De Bruyne B, Pontone G, Patel MR, Norgaard BL, Byrne RA, et al. 1-year outcomes of ffrct-guided care in patients with suspected coronary disease: The PLATFORM study. J Am Coll Cardiol. 2016;68(5):435–445. DOI: 10.1016/j.jacc.2016.05.057 27470449

[11] Curzen N, Nicholas Z, Stuart B, Wilding S, Hill K, Shambrook J, et al. Fractional flow reserve derived from computed tomography coronary angiography in the assessment and management of stable chest pain: the FORECAST randomized trial. Eur Heart J. 2021;42(37):3844–3852. DOI: 10.1093/eurheartj/ehab444 34269376 PMC8648068

[12] Liu X, Mo X, Zhang H, Yang G, Shi C, Hau WK. A 2-year investigation of the impact of the computed tomography-derived fractional flow reserve calculated using a deep learning algorithm on routine decision-making for coronary artery disease management. Eur Radiol. 2021;31(9):7039–7046. DOI: 10.1007/s00330-021-07771-7 33630159

[13] Li Y, Qiu H, Hou Z, Zheng J, Li J, Yin Y, et al. Additional value of deep learning computed tomographic angiography-based fractional flow reserve in detecting coronary stenosis and predicting outcomes. Acta Radiol. 2022;63(1):133–140. DOI: 10.1177/0284185120983977 33423530

[14] Fairbairn TA, Nieman K, Akasaka T, Norgaard BL, Berman DS, Raff G, et al. Real-world clinical utility and impact on clinical decision-making of coronary computed tomography angiography-derived fractional flow reserve: Lessons from the ADVANCE Registry. Eur Heart J. 2018;39(41):3701–3711. DOI: 10.1093/eurheartj/ehy530 30165613 PMC6215963

[15] Zhao N, Gao Y, Xu B, Yang W, Song L, Jiang T, et al. Effect of coronary calcification severity on measurements and diagnostic performance of CT-FFR with computational fluid dynamics: results from CT-FFR CHINA Trial. Front Cardiovasc Med. 2021;8:810625. DOI: 10.3389/fcvm.2021.810625 35047581 PMC8761984

[16] Yang J, Shan D, Wang X, Sun X, Shao M, Wang K, et al. On-site computed tomography-derived fractional flow reserve to guide management of patients with stable coronary artery disease: The TARGET Randomized Trial. Circulation. 2023;147(18):1369–1381. DOI: 10.1161/CIRCULATIONAHA.123.063996 36870065

[17] Chen YD, Fang WY, Chen JY, Fan ZM, Gao CY, Ge JB, et al. Chinese expert consensus on the noninvasive imaging examination pathways of stable coronary artery disease. J Geriatr Cardiol. 2018;15(1):30–40. 29434623 10.11909/j.issn.1671-5411.2018.01.012 PMC5803535

[18] Zhang LJ, Tang C, Xu P, Guo B, Zhou F, Xue Y, et al. Coronary computed tomography angiography-derived fractional flow reserve: An expert consensus document of Chinese Society of Radiology. J Thorac Imaging. 2022;37(6):385–400. DOI: 10.1097/RTI.0000000000000679 36162081

[19] Liao L, Feng H, Jiao J, Zhao Y, Ning H. Nursing assistants’ knowledge, attitudes and training needs regarding urinary incontinence in nursing homes: A mixed-methods study. BMC Geriatr. 2023;23(1):39. DOI: 10.1186/s12877-023-03762-z 36683023 PMC9867858

[20] Andrade C, Menon V, Ameen S, Kumar Praharaj S. Designing and conducting knowledge, attitude, and practice surveys in psychiatry: Practical guidance. Indian J Psychol Med. 2020;42(5):478–481. DOI: 10.1177/0253717620946111 33414597 PMC7750837

[21] World Health Organization. Advocacy, communication and social mobilization for TB control: A guide to developing knowledge, attitude and practice surveys. http://whqlibdoc.who.int/publications/2008/9789241596176_eng.pdf. Accessed November 22, 20222008.

[22] Zarei F, Dehghani A, Ratansiri A, Ghaffari M, Raina SK, Halimi A, et al. ChecKAP: A Checklist for Reporting a Knowledge, Attitude, and Practice (KAP) Study. Asian Pac J Cancer Prev. 2024;25(7):2573–2577. DOI: 10.31557/APJCP.2024.25.7.2573 39068593 PMC11480624

[23] Althubaiti A. Sample size determination: A practical guide for health researchers. J Gen Fam Med. 2023;24(2):72–78. DOI: 10.1002/jgf2.600 36909790 PMC10000262

[24] Islam MZ, Islam MS, Kundu LR, Ahmed A, Hsan K, Pardhan S, et al. Knowledge, attitudes and practices regarding antimicrobial usage, spread and resistance emergence in commercial poultry farms of Rajshahi district in Bangladesh. PLoS One. 2022;17(11):e0275856. DOI: 10.1371/journal.pone.0275856 36378627 PMC9665401

[25] Barua A. Methods for decision-making in survey questionnaires based on Likert scale. J Asian Sci Res. 2013;3(1):35–38.

[26] Nakanishi R, Budoff MJ. Noninvasive FFR derived from coronary CT angiography in the management of coronary artery disease: Technology and clinical update. Vasc Health Risk Manag. 2016;12:269–278. DOI: 10.2147/VHRM.S79632 27382296 PMC4922813

[27] Koo B-K, Erglis A, Doh J-H, Daniels DV, Jegere S, Kim H-S, et al. Diagnosis of ischemia-causing coronary stenoses by noninvasive fractional flow reserve computed from coronary computed tomographic angiograms. Results from the prospective multicenter DISCOVER-FLOW (Diagnosis of Ischemia-Causing Stenoses Obtained Via Noninvasive Fractional Flow Reserve) study. J Am Coll Cardiol. 2011;58(19):1989–1997. DOI: 10.1016/j.jacc.2011.06.066 22032711

[28] Nørgaard BL, Leipsic J, Gaur S, Seneviratne S, Ko BS, Ito H, et al. Diagnostic performance of noninvasive fractional flow reserve derived from coronary computed tomography angiography in suspected coronary artery disease: The NXT trial (Analysis of Coronary Blood Flow Using CT Angiography: Next Steps). J Am Coll Cardiol. 2014;63(12):1145–1155. DOI: 10.1016/j.jacc.2013.11.043 24486266

[29] Tesche C, Vliegenthart R, Duguay TM, De Cecco CN, Albrecht MH, De Santis D, et al. Coronary computed tomographic angiography-derived fractional flow reserve for therapeutic decision making. Am J Cardiol. 2017;120(12):2121–2127. DOI: 10.1016/j.amjcard.2017.08.034 29102036

[30] Tang CX, Liu CY, Lu MJ, Schoepf UJ, Tesche C, Bayer RR, et al. CT FFR for ischemia-specific cad with a new computational fluid dynamics algorithm: A Chinese Multicenter study. JACC Cardiovasc Imaging. 2020;13(4):980–990. DOI: 10.1016/j.jcmg.2019.06.018 31422138

[31] Tesche C, Otani K, De Cecco CN, Coenen A, De Geer J, Kruk M, et al. Influence of coronary calcium on diagnostic performance of machine learning CT-FFR: Results from MACHINE Registry. JACC Cardiovasc Imaging. 2020;13(3):760–770. DOI: 10.1016/j.jcmg.2019.06.027 31422141

[32] Li M, Zhou T, Yang L-f, Peng Z-h, Ding J, Sun G. Diagnostic accuracy of myocardial magnetic resonance perfusion to diagnose ischemic stenosis with fractional flow reserve as reference: Systematic review and meta-analysis. JACC Cardiovasc Imaging. 2014;7(11):1098–1105. DOI: 10.1016/j.jcmg.2014.07.011 25306540

[33] Coenen A, Kim Y-H, Kruk M, Tesche C, De Geer J, Kurata A, et al. Diagnostic accuracy of a machine-learning approach to coronary computed tomographic angiography-based fractional flow reserve: Result from the MACHINE Consortium. Circ Cardiovasc Imaging. 2018;11(6):e007217. DOI: 10.1161/CIRCIMAGING.117.007217 29914866

[34] Danad I, Szymonifka J, Twisk JWR, Norgaard BL, Zarins CK, Knaapen P, et al. Diagnostic performance of cardiac imaging methods to diagnose ischaemia-causing coronary artery disease when directly compared with fractional flow reserve as a reference standard: A meta-analysis. Eur Heart J. 2017;38(13):991–998. DOI: 10.1093/eurheartj/ehw095 27141095 PMC5381594

[35] Burch RA, Siddiqui TA, Tou LC, Turner KB, Umair M. The cost effectiveness of coronary ct angiography and the effective utilization of CT-fractional flow reserve in the diagnosis of coronary artery disease. J Cardiovasc Dev Dis. 2023;10(1):25. DOI: 10.3390/jcdd10010025 36661920 PMC9863924

[36] Muhummad Sohaib N, Yael R-G, Tiago R, Khan Ha B, Anna Buylova G, Amedeo C, et al. Cost-effectiveness in diagnosis of stable angina patients: A decision-analytical modelling approach. Open Heart. 2022;9(1):e001700. DOI: 10.1136/openhrt-2021-001700 35379740 PMC8981340

[37] Fairbairn TA, Mullen L, Nicol E, Lip GYH, Schmitt M, Shaw M, et al. Implementation of a national AI technology program on cardiovascular outcomes and the health system. Nature Medicine. 2025;31(6):1903–1910. DOI: 10.1038/s41591-025-03620-y PMC12176617 40186078

[38] Fujimoto S, Nozaki YO, Sakamoto T, Nakanishi R, Asano T, Kadota K, et al. Clinical impacts of CT-derived fractional flow reserve under insurance reimbursement: Results from multicenter, prospective registry. J Cardiol. 2024;84(2):126–132. DOI: 10.1016/j.jjcc.2023.11.002 37949315

[39] Matsuo H, Kawasaki T, Amano T, Kawase Y, Sobue Y, Kondo T, et al. Effect of coronary computed tomography angiography-derived fractional flow reserve on physicians’ clinical behavior- differences between sites with and without appropriate use criteria as designated by the Japanese Reimbursement System. Circ Rep. 2020;2(7):364–371. DOI: 10.1253/circrep.CR-20-0038 33693254 PMC7932815

[40] O’Leary RA, Burn J, Urwin SG, Sims AJ, Beattie A, Bagnall A. Impact on stable chest pain pathways of CT fractional flow reserve. Heart. 2023;109(18):1380–1386. DOI: 10.1136/heartjnl-2022-321923 37080766 PMC10511976

[41] Williams AE, Croft J, Napp V, Corrigan N, Brown JM, Hulme C, et al. SaFaRI: Sacral nerve stimulation versus the FENIX magnetic sphincter augmentation for adult faecal incontinence: a randomised investigation. Int J Colorectal Dis. 2016;31(2):465–472. DOI: 10.1007/s00384-015-2492-3 26754071 PMC4744248

[42] Liyew B, Dejen Tilahun A, Kassew T. Knowledge, attitude, and associated factors towards physical assessment among nurses working in intensive care units: A multicenter cross-sectional study. Crit Care Res Pract. 2020;2020:9145105. DOI: 10.1155/2020/9145105 32850150 PMC7436285

[43] Zims H, Karay Y, Neugebauer P, Herzig S, Stosch C. Fifteen years of the cologne medical model study course: has the expectation of increasing student interest in general practice specialization been fulfilled? GMS J Med Educ. 2019;36(5):Doc58. 31815168 10.3205/zma001266 PMC6883249

[44] Cavallo F, Esposito R, Limosani R, Manzi A, Bevilacqua R, Felici E, et al. Robotic services acceptance in smart environments with older adults: User Satisfaction and Acceptability Study. J Med Internet Res. 2018;20(9):e264. DOI: 10.2196/jmir.9460 30249588 PMC6231879

[45] Ahmed I, Sasikumar N. Echocardiography Imaging Techniques. StatPearls. Treasure Island, FL: Ineligible companies. 34283496

[46] Althubaiti A. Information bias in health research: Definition, pitfalls, and adjustment methods. J Multidiscip Healthc. 2016;9:211–217. DOI: 10.2147/JMDH.S104807 27217764 PMC4862344

[47] Blumstein DT, Williams DM, Lim AN, Kroeger S, Martin JGA. Strong social relationships are associated with decreased longevity in a facultatively social mammal. Proc Biol Sci. 2018;285(1871). DOI: 10.1098/rspb.2017.1934 PMC5805924 29343594

