# Supplementary material for: Attitudes and Willingness of Cardiothoracic Group Physicians in the Cardiovascular and Radiology Departments toward the Adjuvant Use of CT-Derived Fractional Flow Reserve in the Diagnosis of Coronary Artery Disease
Source: Glob Heart. 2025 Oct 6;20(1):87. doi: 10.5334/gh.1477 (PMC12513361; doi:10.5334/gh.1477)
Supplement: Supplementary File. — Tables S1 to S3. [file gh-20-1-1477-s1.pdf]

Table S1 Participants' demographic information

	N(%)	Attitude Dimension Median (25th percentile, 75th percentile)	P	Will Dimension Median (25th percentile, 75th percentile)	P
**Total**	265	51(48, 55)		31(29, 32)	
**Gender**			0.834		0.244
Male	151(57.0)	51(48, 54)		31(29, 32)	
Female	114(43.0)	51(48, 55)		31(28, 32)	
**Age**			0.853		0.441
Below 30 years	37(14.0)	51(48, 55)		32(29, 32)	
31~40 years	120(45.3)	51(48, 54)		31(29, 32)	
41~50 years	83(31.3)	51(47, 55)		31(28, 32)	
Above 50 years	25(9.4)	51(49, 56)		30(29, 32)	
**Marital Status**			0.792		0.892
Married	221(83.4)	51(48, 55)		31(28, 32)	

Unmarried	42(15.8)	51(48, 55)		31(29, 32)	
Divorced	2(0.8)	49(42, 56)		29.5(27, 32)	
Widowed	0(0.0)	/		0(0.0)	/
**Education Level**			0.262		0.166
Junior College and Belove	10(3.8)	51(49, 53)		30(28, 31)	
Bachelor’s Degree	93(35.1)	51(47, 54)		31(28, 32)	
Graduate Degree	162(61.1)	51(49, 55)		31(29, 32)	
**Occupation Type**			0.509		0.333
Physician	242(91.3)	51(48, 55)		31(28, 32)	
Technician	18(6.8)	51(50, 53)		31(29, 32)	
Nurse	5(1.9)	52(52, 54)		32(32, 32)	
**Title**			0.596		0.459
None	9(3.4)	50(46, 51)		30(26, 32)	
Junior	44(16.6)	51(48.5, 54)		31.5(28.5, 33)	
Intermediate	127(47.9)	51(48, 55)		31(29, 32)	
Associate Senior	63(23.8)	51(47, 55)		30(27, 32)	

Senior	22(8.3)	51.5(49, 56)		32(30, 32)	
**Type of Medical Institution**			0.243		0.336
General Hospital	172(64.9)	51(48, 55)		31(29, 32)	
Specialized Hospital	23(8.7)	49(46, 52)		31(28, 36)	
Traditional Chinese Medicine Hospital	58(21.9)	51(49, 54)		30.5(29, 32)	
Other	12(4.5)	49.5(48.5, 54)		28.5(27, 32)	
**Nature of Workplace**			0.830		0.749
Public Tier 1 Hospital	14(5.3)	52(49, 56)		29.5(28, 32)	
Public Tier 2 Hospital	30(11.3)	52(49, 54)		31(29, 32)	
Public Tier 3 Hospital	212(80.0)	51(48, 55)		31(29, 32)	
Private Hospital	9(3.4)	51(48, 54)		31(30, 33)	
**Work Experience**			0.055		0.353
Below 1 year	8(3.0)	47(43.5, 49)		28.5(24, 31)	
1-3 years	32(12.1)	51(48.5, 54.5)		30.5(29, 33)	
4-6 years	35(13.2)	51(48, 55)		31(29, 32)	

7-10 years	45(17.0)	51(48, 54)		31(30, 32)	
Above 10 years	145(54.7)	51(49, 55)		31(28, 32)	
**Number of Activity Participations per Year**			0.196		0.542
Less than 2 times	41(15.5)	51(47, 54)		31(29, 32)	
2-5 times	107(40.4)	51(47, 54)		31(28, 32)	
6-10 times	61(23.0)	52(50, 55)		31(28, 32)	
More than 10 times	56(21.1)	50.5(49, 54)		32(29, 33)	

Table S2 Attitude Dimension Score distribution

Attitude Dimension Items	N(%)	Median (25th percentile, 75th percentile)	P
**1. How well do you understand CT-derived fractional flow reserve (CT-FFR) technology?**			< 0.001
1 or 2 points	52(19.6)	47.5(44, 50.5)	
4 or 5 points	116(43.8)	53.5(50, 57)	
**7. Compared to conventional invasive wire-based FFR, do you worry about the accuracy of CT-derived fractional flow reserve (CT-FFR) results?**			< 0.001
1 or 2 points	128(48.3)	50(47, 52)	
4 or 5 points	36(13.6)	56(53.5, 63)	
**8. Are you concerned that inaccurate CT-derived fractional flow reserve (CT-FFR) results may lead to medical litigation?**			< 0.001
1 or 2 points	100(37.7)	49(46, 52)	
4 or 5 points	53(20.0)	55(53, 63)	

**15. Do you think it will take a considerable amount of time for the widespread adoption of CT-derived fractional flow reserve (CT-FFR) technology?**			< 0.001
1 or 2 points	180(67.9)	51(48, 54)	
4 or 5 points	15(5.7)	63(51, 67)	

Table S3 Attitude and willingness dimension

Attitude Dimension

**Cut-off Value：**<br><br>**≥51 / < 51**		**Single factor**		**Multi-factor（Regression method = input）**	
	**No.**	**OR(95%CI)**	**P**	**OR(95%CI)**	**P**
**Gender**					
Male	85/151	ref.			
Female	61/114	0.894(0.548, 1.457)	0.652		
**Gender**					
Below 30 years	20/37	ref.			
31-40 years	65/120	1.005(0.479, 2.105)	0.990		
41-50 years	47/83	1.110(0.509, 2.418)	0.793		
Above 50 years	14/25	1.082(0.390, 3.002)	0.880		
**Marital Status**					
Other	25/44	ref.			

Married	121/221	0.920(0.479, 1.766)	0.801		
**Education Level**					
Bachelor’s degree and below	54/103	ref.			
Graduate	92/162	1.193(0.726, 1.959)	0.487		
**Occupation Type**					
Other	18/23	ref.		ref.	
Physician	128/242	0.312(0.112, 0.867)	0.026	0.343(0.123, 0.957)	0.041
**Title**					
None and Junior title	30/53	ref.			
Intermediate	66/127	0.830(0.435, 1.581)	0.570		
Associate Senior	35/63	0.958(0.459, 2.001)	0.910		
Senior	15/22	1.643(0.576, 4.689)	0.354		
**Medical Institution Type**					
Other Hospitals	139/242	ref.		ref.	
Specialist Hospitals	7/23	0.324(0.129, 0.817)	0.017	0.354(0.140, 0.896)	0.028
**Nature of Workplace**					

Other Hospitals	34/53	ref.	
Public Tertiary Hospitals	112/212	0.626(0.336, 1.167)	0.140
**Work Experience**			
Below 3 years	18/40	ref.	
4-6 years	18/35	1.294(0.521, 3.214)	0.579
7-10 years	24/45	1.397(0.594, 3.284)	0.444
Above 10 years	86/145	1.782(0.880, 3.608)	0.109
**Number of Activity Participations per Year**			
Less than 2 times	23/41	ref.	
2-5 times	56/107	0.859(0.417, 1.773)	0.682
6-10 times	39/61	1.387(0.618, 3.114)	0.427
More than 10 times	28/56	0.783(0.348, 1.758)	0.553

Willingness Dimension

**Cut-off Value：**	**Single factor**	**Multi-factor**

≥31 / < 31

	No.	OR(95%CI)	P	OR(95%CI)	P
**Attitude dimension score**					
Low (<51)	37/118	ref.		ref.	
High (≥51)	109/147	6.280(3.673, 10.736)	< 0.001	6.280(3.673, 10.736)	< 0.001
**Gender**	109/147				
Male	86/151	ref.			
Female	61/114	0.870(0.533, 1.419)	0.576		
**Gender**					
Below 30 years	22/37	ref.			
31-40 years	71/120	0.988(0.466, 2.092)	0.975		
41-50 years	42/83	0.698(0.319, 1.531)	0.370		
Above 50 years	12/25	0.629(0.226, 1.750)	0.375		
**Marital Status**					
Other	23/44	ref.			

Married	124/221	1.167(0.610, 2.232)	0.640
**Education Level**			
Bachelor’s degree and below	52/103	ref.	
Graduate	95/162	1.391(0.846, 2.286)	0.193
**Occupation Type**			
Other	15/23	ref.	
Physician	132/242	0.640(0.262, 1.566)	0.328
**Title**			
None and Junior title	32/53	ref.	
Intermediate	71/127	0.832(0.433, 1.598)	0.581
Associate Senior	30/63	0.597(0.285, 1.250)	0.171
Senior	14/22	1.148(0.411, 3.212)	0.792
**Medical Institution Type**			
Other Hospitals	134/242	ref.	
Specialist Hospitals	13/23	1.048(0.442, 2.482)	0.916
**Nature of Workplace**			

Other Hospitals	30/53	ref.	
Public Tertiary Hospitals	117/212	0.944(0.515, 1.732)	0.853
**Work Experience**			
Below 3 years	18/40	ref.	
4-6 years	19/35	1.451(0.584, 3.610)	0.423
7-10 years	29/45	2.215(0.926, 5.299)	0.074
Above 10 years	81/145	1.547(0.765, 3.127)	0.224
**Number of Activity Participations per Year**			
Less than 2 times	25/41	ref.	
2-5 times	56/107	0.703(0.338, 1.463)	0.346
6-10 times	32/61	0.706(0.316, 1.578)	0.396
More than 10 times	34/56	0.989(0.433, 2.258)	0.979
